# Digital education in preclinical prosthodontics: a critical review of recent evidence

**DOI:** 10.1038/s41405-026-00453-w

**Published:** 2026-06-19

**Authors:** Nissaf Daouahi, Yosra Gassara

**Affiliations:** Department of Fixed Prosthodontics, Faculty of Dentistry, University of Monastir, Monastir, Tunisia

**Keywords:** Dentistry, Dental education, Dental clinical teaching

**To the Editor**,

We read with great interest the study by Lan-Anh Thi Pham et al. [1] recently published in BDJ Open (10.1038/s41405-025-00344-6), which evaluated the effectiveness of a digital educational system on crown preparation performance in preclinical fixed prosthodontic training [1]. The integration of digital assessment software in preclinical education represents an important advancement in dental pedagogy. Nevertheless, several methodological considerations merit discussion.

**First**, the study included 40 third-year dental students from a single institution. While the sample size exceeds the minimum estimated by the authors’ paired design formula, it remains relatively small for broad generalization. The absence of a parallel control group limits the ability to isolate the effect of digital feedback from improvements attributable to repeated practice. The observed gains in tooth preparation scores may partially reflect the intrinsic learning curve of preclinical students [2].

**Second**, the improvements were primarily noted in occlusal surfaces and overall scores, while axial and proximal surface gains were more variable. This finding aligns with previous literature indicating that certain tooth surfaces are inherently more challenging to prepare and evaluate, even with digital assistance [3]. The study design, which included a final session without digital support, revealed that students’ autonomous performance did not consistently match prior digitally assisted practice, highlighting that digital software, while beneficial, cannot fully replace instructor guidance [1].

**Third**, while the study leveraged typodonts and preclinical simulation to facilitate skill acquisition, these models cannot fully replicate the complexities of the clinical environment, including soft tissue management, patient variability, and restricted oral access, leaving the translation of improved phantom-based performance to real patient care untested. Moreover, although digital measurements demonstrated good intra-rater reliability (ICC = 0.87), the scoring system lacked external validation against established prosthodontic assessment standards, highlighting the need for future studies to undertake a comprehensive psychometric evaluation to strengthen the clinical relevance and generalizability of these findings [4].

**Significantly**, Lan-Anh Thi Pham et al. demonstrate that the Dental Teacher software enhances preclinical crown preparation performance and fosters self-directed learning among dental students. Although the study’s single-institution design, modest sample size, lack of a control group, and reliance on preclinical simulations warrant cautious interpretation, the findings remain highly relevant. Importantly, this work exemplifies the type of high-quality, evidence-based research that BDJ Open continues to publish, reinforcing its role as a leading platform for advancing innovations in dental education. Future studies with larger, multi-institutional cohorts, controlled designs, and clinical performance outcomes will be essential to fully establish the pedagogical impact of digital educational systems.
